# Estimating Total Quantitative Protein Content in *Escherichia coli*, *Saccharomyces cerevisiae*, and HeLa Cells

**DOI:** 10.3390/ijms24032081

**Published:** 2023-01-20

**Authors:** Georgii V. Dolgalev, Taras A. Safonov, Viktoriia A. Arzumanian, Olga I. Kiseleva, Ekaterina V. Poverennaya

**Affiliations:** 1 Institute of Biomedical Chemistry, Moscow 119281, Russia; 2 X-BIO Institute, University of Tyumen, 6 Volodarskogo St., Tyumen 625003, Russia

**Keywords:** quantitative proteomics, absolute protein abundance, total protein, *E. coli*, *S. cerevisiae*, HeLa

## Abstract

The continuous improvement of proteomic techniques, most notably mass spectrometry, has generated quantified proteomes of many organisms with unprecedented depth and accuracy. However, there is still a significant discrepancy in the reported numbers of total protein molecules per specific cell type. In this article, we explore the results of proteomic studies of *Escherichia coli*, *Saccharomyces cerevisiae*, and HeLa cells in terms of total protein copy numbers per cell. We observe up to a ten-fold difference between reported values. Investigating possible reasons for this discrepancy, we conclude that neither an unmeasured fraction of the proteome nor biases in the quantification of individual proteins can explain the observed discrepancy. We normalize protein copy numbers in each study using a total protein amount per cell as reported in the literature and create integrated proteome maps of the selected model organisms. Our results indicate that cells contain from one to three million protein molecules per µm^3^ and that protein copy density decreases with increasing organism complexity.

## 1. Introduction

The difference in observed phenotypes between cells of different organisms and between individual cells of multicellular organisms can be largely explained by differences in their proteome composition [1]. However, the proteome remains much more difficult to quantify than the genome or the transcriptome since it is significantly more complex—the number of different proteoforms in a typical human cell is estimated to be well into the hundreds of thousands or even millions, and individual proteins can exhibit extremely dynamic behavior [2,3]. Additionally, in contrast to genomics, proteomics currently lacks methods for amplifying the signal from proteins present in low numbers, which makes their quantification difficult [4].

Despite all the challenges, the field of proteomics has demonstrated great progress in the quantification of proteomes of different organisms. This progress is, for the most part, attributed to advancements in mass spectrometry that now has sufficient power to quantify tens of thousands of individual proteins in a single experiment [5]. This has resulted in the publication of hundreds of high-quality proteomes of various cell types [6] or even whole tissues [7]. Several alternative approaches to mass spectrometry have also been developed for the purpose of proteome quantification, for instance, fluorescence-based quantification [8], immunoblotting [9] and ribosomal profiling [10]. However, in contrast to mass spectrometry, these methods are less universal. One of the most interesting questions of quantitative proteomics is how many protein molecules in total are present per particular cell type. This question can be approached in two different ways. The first way is to measure the total protein mass per cell and average protein mass, which allows one to calculate an approximate number of proteins per cell by simple division of these two values. Since this calculation requires relatively simple biochemical measurements, such an approach was used well before the development of robust strategies for whole proteome quantification [11]. Another way to approach this question is to quantify levels of individual proteins, and then sum them up to obtain a total protein copy number per cell, which is where mass spectrometry is the method of choice. 

A rigorous attempt to combine and contrast these two approaches was conducted by R. Milo in 2013 [12]. Surprisingly, estimates of the total number of protein copies calculated using protein density per cell and average protein mass contrasted with estimates of total protein copy number from whole proteome quantification experiments. This difference could be as high as ten fold for some cell types. Since the publication of the article in 2013, a plethora of new whole proteome quantification datasets, as well as updated measurements of physiological parameters of various cell types, have been published. This opens an opportunity to explore the proteomes of common model organisms more rigorously and to arrive at consensus values of total protein copies per cell, as well as copy numbers of individual proteins.

## 2. Results

### 2.1. Overview of Selected Proteomic Studies of E. coli, S. cerevisiae, and HeLa Cells

To achieve our goal of understanding how many protein molecules were present per cell, we chose three well studied model organisms for research: *E. coli* as an example of a relatively simple prokaryotic cell, *S. cerevisiae* as a unicellular eukaryote organism, and HeLa cell line as a cell from a complex multicellular organism such as human.

For the chosen cell types, we searched for published proteomic studies that reported copies per cell for these model organisms. We only selected studies that quantified a significant portion of the proteome and performed original calculations of protein copy numbers. In addition, we also required that the studies use similar growth conditions for particular cell types. In total, we found 21 such studies, with 7 studies for each model organism (Table 1). These studies primarily utilized shotgun mass spectrometry. Additionally, one study by Lawless et al. performed targeted protein quantification using the single reaction monitoring (SRM) technique for an impressive number of proteins (>1000) [13]. While mass spectrometry is the method of choice for proteome quantification, three of the selected studies utilized alternative methods such as immunoblotting (Ghaemmaghami et al., 2003 [9]), single molecule fluorescence-based protein abundance quantification (Taniguchi et al., 2010 [8]), and ribosomal profiling (Li et al., 2014 [10]).

To obtain copies per cell, the first step in proteomic studies is to derive absolute abundance values for each quantified protein, i.e., values that are directly proportional to protein concentration in the cell. This is the most important step for studies that use mass spectrometry. As peptides have different efficiencies of ionization, their individual abundance alone cannot be directly converted to protein abundance [4]. A classic way to circumvent this problem is to spike-in labeled peptides or proteins with known concentration, which allows us to infer the absolute abundance of their unlabeled counterparts by comparison [29]. However, this is an extremely expensive and laborious approach if more than a few proteins have to be quantified, and only one study in our selection performed such a procedure for all proteins, which was by Lawless et al., 2016 [13]. However, most of the other studies opted for a label-free quantification approach, which typically consisted of inferring absolute protein abundance based on some form of integration of peptide-level data. Such approaches, for example, total protein approach (TPA) [15] or intensity-based absolute quantification (iBAQ) [30] have important technical differences but are nonetheless considered to be similar for our goals. Regarding the studies that did not utilize mass spectrometry, Western blotting and fluorescence only require calibration with standards of known quantity, although certain care must be taken when converting chemiluminescence/fluorescence intensity to protein abundance [31]. One remaining study by Li et al. utilized ribosomal profiling to estimate absolute protein synthesis rates that were directly proportional to protein abundance if the influence of post-translational control on protein levels was negligible, as was true for *E. coli* cells [10].

The second step to obtaining protein copy numbers consists of converting absolute abundance values to protein copies per cell, which is required for mass spectrometry and other approaches alike. Again, at this step, there is significant difference in possible approaches. The first approach is to introduce a limited set of labeled proteins into the sample and build a calibration curve, thus, inferring protein abundances in copies/moles in the sample, which was done, for instance, by Schmidt et al. [6]. After this step, however, to obtain protein copy numbers per cell, cell counts that were used as input must be measured, either by flow cytometry or plate counting. Alternatively, if the total protein content in grams per cell is known, absolute abundance values can be converted to protein copies by normalization of the sum of intensities to total protein per cell. One other approach, termed “proteomic ruler” relies on the fact that the number of histones associated with DNA is constant for a specific type of eukaryotic cell, and thus, protein copies can be calculated by normalizing the summed intensity of histones to the protein/DNA ratio per cell [32]. To summarize, selected proteomic studies of *E. coli*, *S. cerevisiae*, and HeLa cells utilize various multi-step approaches to estimate the number of protein copies per cell, and discrepancies at each step can lead to a compound effect on the difference in copy numbers of individual proteins and, thus potentially, total protein copy number per cell.

### 2.2. Comparison of Reported Total Protein Copy Numbers for Selected Model Organisms

**Figure 1:**
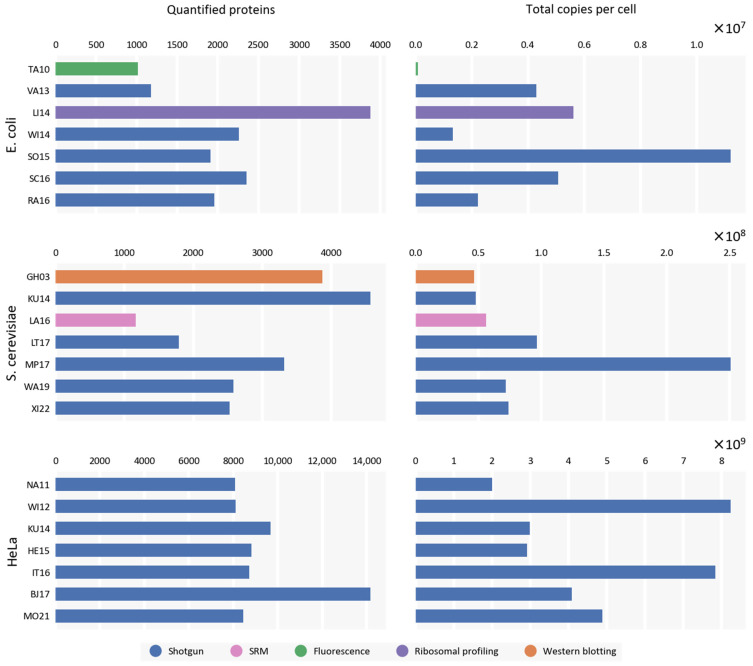
Results of proteomic studies in terms of the number of quantified proteins and total protein copies per cell for *E. coli*, *S. cerevisiae*, and HeLa.

In terms of quantified proteins, studies have quantified variable numbers of them, ranging from 1000 to almost 14,000 proteins (Figure 1). For each of the selected studies, we extracted unadjusted values of protein copies per cell and summed them to obtain the total number of protein copies per cell for each study. The results for each model organism demonstrate significant discrepancies in terms of reported total protein copy numbers (Figure 1).

As expected, there is no correlation observed between the number of quantified proteins in a particular study and the calculated number of total protein copies per cell for each model organism (Pearson’s r = 0.28 for *E. coli*, 0.05 for *S. cerevisiae*, and −0.26 for HeLa). Consequently, some sort of normalization procedure is required before the results of these studies can be integrated into a global proteomic map of the selected model organisms, thus, obtaining a consensus value of total protein copies per cell for a particular model organism and condition.

### 2.3. Exploration of Possible Reasons for the Discrepancy in Reported Total Protein Copy Numbers

To enable cross-study comparisons, first, we mapped all identifiers in the study to the universal UniProt IDs from the most recent release of the UniProt database. As a result, some entries were lost due to possible nomenclature updates or because it was a protein group, rather than a single protein that was quantified. However, despite these losses, our procedure led to the assignment of >95% of all identifiers in most of the studies and led to no more than 10% loss of protein copies reported, which was negligible for our needs (Table S1).

To understand the source of the observed differences, we considered whether proteomic studies can omit a certain part of the proteome from identification due to method-specific biases. Since shotgun mass spectrometry and ribosomal profiling represent untargeted approaches to protein identification, biases in protein detection can leave a certain part of the proteome undetected, depending on the specific method employed. In contrast, targeted approaches such as Western blotting and fluorescence imaging can omit certain proteins, which may have high copy numbers, from the initial selection of targets altogether.

**Figure 2:**
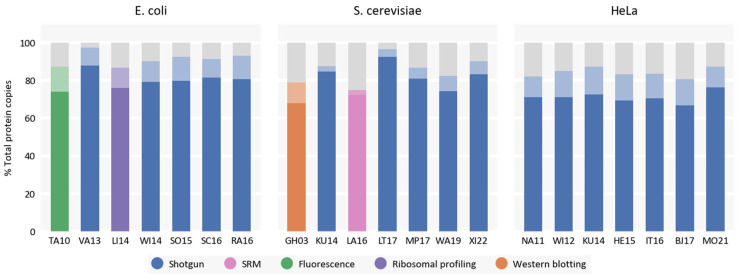
Contribution of proteins quantified in all untargeted datasets for a particular model organism (core proteins) to the reported total protein copy numbers as a percent of total copies. Less saturated portions of the bars represent contributions of proteins which are present in at least n − 1 untargeted dataset for a particular model organism (core 1) to total protein copies.

To analyze the extent of this “hidden proteome”, we quantified how proteins identified in all untargeted studies (core proteins) contributed to the reported total protein copy numbers. We additionally excluded a study by Lahtvee et al. for *S. cerevisiae* from the calculation of the core because this study quantified significantly fewer proteins than in other untargeted studies of *S. cerevisiae* proteome, and exclusion of this dataset improved the contribution of core proteins to the total copy numbers, which was not true when excluding one of any other untargeted studies. We identified 934 proteins present in all untargeted studies of *E. coli* proteome (22% of all predicted protein-coding genes [33]), 1945 core proteins for *S. cerevisiae* cells (32% of all predicted protein-coding genes [34]), and 5051 proteins (24% of predicted number of human protein-coding genes [35]) for HeLa cells. Despite representing from a quarter to a third of all quantified proteins in each study, core proteins account for more than 70–80% of total protein copies per cell for all model organisms (Figure 2). If we relax our definition of core proteins to include proteins present in at least n−1 study (core 1), the contribution of such proteins increases to almost more than 90% of total protein copies per cell for the majority of studies (Figure 2). Thus, we reason that the most frequently detected proteins contribute to the majority of overall copies irrespective of the study. Additionally, we observe that untargeted studies quantify not all of the core proteins (Table S1), which may be the reason for the lower estimates of total protein copy numbers in these datasets.

**Figure 3:**
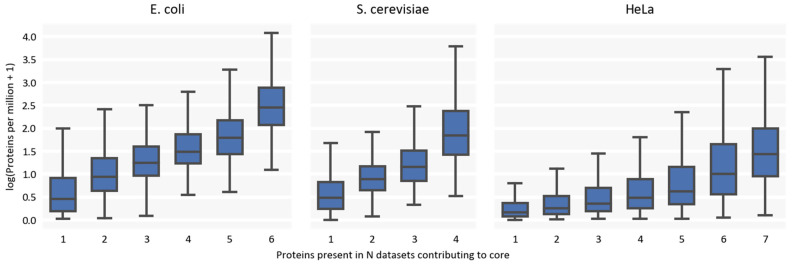
Normalized expression of most and least commonly detected proteins. Proteins per million denote protein copy number divided by the sum of all protein copies in the dataset and multiplied by a million.

Nevertheless, it is unlikely but possible that some systematic bias prevents all studies from detecting a certain part of the proteome. To estimate the contribution of this hypothetical part of the proteome to the overall numbers, we analyzed how the frequency of detection of proteins (as being present in a certain number of datasets) correlates with their averaged normalized expression (Figure 3). The results indicate that there is a clear trend of underrepresentation of proteins with low biological abundance. Therefore, the fraction of unseen proteins likely represents proteins with very low expression values that cannot have such a profound effect on the total protein copy numbers.

**Figure 4:**
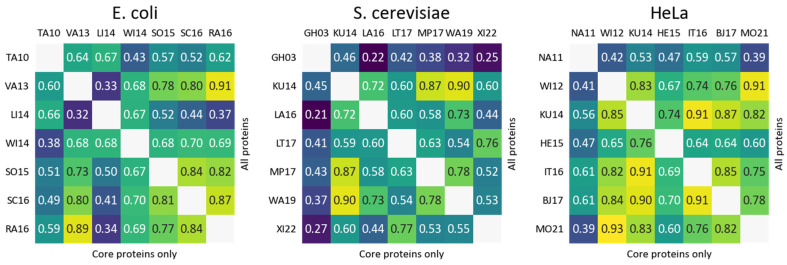
Pairwise Pearson’s correlation of protein levels between individual datasets for all shared proteins (**top right half**) or core proteins (**lower left half**).

To explore the possible discrepancies in quantification of individual proteins, we calculated pairwise Pearson’s correlation for selected datasets (Figure 4). The overall levels of correlation are moderate (mean Pearson’s r = 0.63 for *E. coli*, 0.57 for *S. cerevisiae*, and 0.70 for Hela cells). Moderate levels of pairwise correlations are typical for mass spectrometry-based experiments, although, of course, a better level is expected since identical cell types in almost identical conditions are considered. Interestingly, a targeted immunoblot-based study of *S. cerevisiae* by Ghaemmaghami et al. demonstrated lower-than-average levels of correlation with mass spectrometry studies. Additionally, a fluorescence-based study of the *E. coli* proteome by Taniguchi et al. showed a moderate level of correlation with other datasets despite quantifying a significantly smaller number of proteins in total. It is also surprising that we do not observe an increased level of correlation when taking into consideration only core proteins (lower left corner of heatmaps in Figure 4).

In conclusion, we reason that although abundances of specific proteins can be the reason for some of the discrepancies observed between total protein copy numbers, this factor alone cannot explain a ten-fold difference in some cases. Therefore, we believe that neither the unidentified portion of the proteome nor differences in quantification of particular proteins can produce the observed effect. In turn, it is likely that some sort of a study-specific coefficient can be applied to normalize quantitative information between different studies of a particular model organism.

### 2.4. Estimation of Total Protein Copy Numbers per Cell from Total Protein Mass per Cell

To produce consensus values of protein abundances per cell, we opted for a simple, but powerful approach of estimating the total number of protein copies per cell based on the total protein mass per cell and average protein mass.

**Figure 5:**
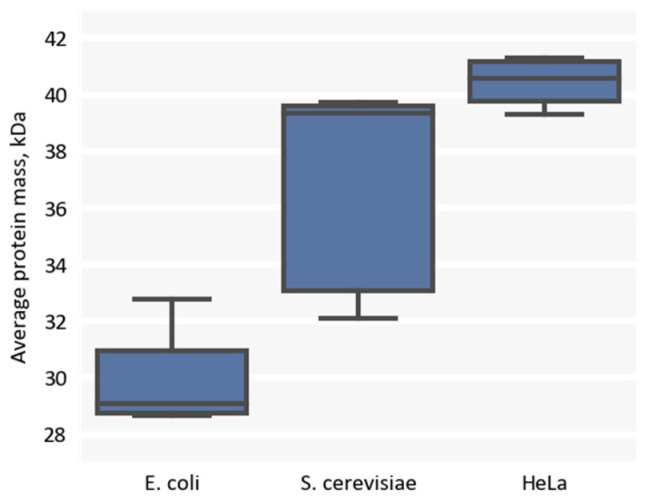
Distribution of average protein masses in the selected proteomic studies of *E. coli*, *S. cerevisiae*, and HeLa.

As our first step, we calculated average protein molecular masses as reported in the datasets (Figure 5). Our results aligned with the well established observation that prokaryotes have a lower average protein mass (around 30 kDa) than eukaryotes (around 40 kDa) [12], although we also noticed a small difference in the median protein mass between *S. cerevisiae* and HeLa cells, which may have reflected the relative extent of cell complexity between yeast and humans.

**Figure 6:**
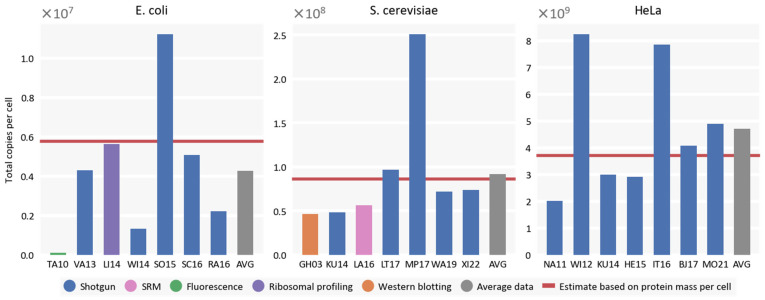
Comparisons of reported numbers of total protein copies per cell and the average values with estimates from total protein mass per cell.

Next, we analyzed the reports of measured total protein weight per cell of *E. coli*, *S. cerevisiae*, and HeLa cells. We selected close to average measurements that were preferentially obtained in recent years. For *E. coli* cells, we took the value of 280 ng per cell, obtained by Schmidt et al. for our conditions of interest [6]. For *S. cerevisiae*, values from 4.9 to 6.4 pg were reported and we selected the average, i.e., 5.65 pg [36]. For HeLa cells, values from 200 to 300 pg were reported [18,37]. We took the average of these values for our calculations. Our selected values differed slightly from those taken by Milo [12]. Next, we estimated the total number of protein copies per cell by simply dividing the total protein mass per cell by the median average protein mass obtained for the selected cell types. We obtained the following numbers of total protein copies per cell: 5,774,718 for *E. coli*, 86,196,924 for *S. cerevisiae*, and 3,698,713,400 for HeLa cells. Comparing the obtained results with reported protein copy numbers from the selected studies, we noticed that our estimates were close to the average of reported values (Figure 6). Therefore, we reasoned that it was correct to normalize reported protein copies per cell to our calculated values for the chosen model organisms.

To enable direct comparisons of the cellular proteome numbers between such different organisms, we converted the values of total protein copies per cell to total protein copies per unit of volume, µm^3^, by dividing our estimates by a typical cellular volume in conditions typical for the selected studies. While the measurements of cell volume for *S. cerevisiae* (42 µm^3^ [38,39]) and for HeLa cells (2425 µm^3^ [40]) are fairly consistent in the available literature, reported values for *E. coli* cells display significant discrepancy, ranging from 1 µm^3^ to almost 3 µm^3^ in similar growth conditions [17,41,42,43]. *E. coli* and bacteria, in general, have a well documented phenomenon of demonstrating significant changes in cell volume depending on the growth rate. However, this is unlikely to explain the observed discrepancy as the aforementioned estimates were calculated for cells growing in similar conditions, and thus, with a similar growth rate. As a consensus value, we selected 2.15 µm^3^, reported by Radzikowski et al. [17] (see also supplementary note in Schmidt et al. [6]). For the selected cell volumes, we obtained values of 2,685,915 protein copies per µm^3^ for *E. coli*, 2,034,146 protein copies per µm^3^ *S. cerevisiae*, and 1,525,243 protein copies per µm^3^ for HeLa. As can be seen, there is a clear trend of decreasing protein copies per unit of cell volume with increasing organism complexity, although it is not as profound as estimated previously [12].

### 2.5. Normalization and Integration of Protein Copy Numbers in the Selected Studies

To enable the normalization of protein copy numbers according to a calculated total number of copies per cell, we developed and applied a normalization procedure to all datasets. Since individual studies quantified different numbers of proteins in total, simply normalizing the sum of all protein copies to an estimated number of total copies per cell would yield relatively increased values for datasets that quantified lower numbers of proteins. Accordingly, we decided to base our normalization procedure on the contribution of core proteins to the number of total copies. For each cell type, we calculated a minimal contribution of core proteins to total copy numbers across untargeted studies. Then, we normalized the sum of all core protein copies in each dataset to our estimated total number of protein copies per cell multiplied by the minimal observed contribution of core proteins to the total copy number. For untargeted studies, we also multiplied an estimated sum of core protein copy numbers by a proportion of core proteins that were detected in the untargeted study. Finally, we calculated the average copy number for each detected protein, thus, obtaining an integrated proteome for each cell type (Table S3). Our data include estimated copy number values for 3852 proteins in *E. coli* (91% of all predicted protein-coding genes [33]), 4680 proteins for *S. cerevisiae* (77% of all predicted protein-coding genes [34]), and 12,653 proteins for HeLa (60% of all predicted protein-coding genes [35]).

The sums of our averaged results are, as expected, close to the calculated number of proteins per cell type, differing no more than 10% from initial estimates (5,852,319 for E. coli, 81,627,580 for S. cerevisiae, and 3,360,824,528 for HeLa cells). Small differences can be explained by discrepancies in quantification of individual proteins, plus the fact that not all predicted proteins of the model organisms are present in the resulting proteomes. Additionally, our results correlate well with a previously published integrated analysis of *S. cerevisiae* proteome partly based on the datasets also selected for our study (Pearson’s r = 0.82) [44]; however, our results are almost twice as high in terms of total protein copies per cell despite more proteins being included in the published dataset. In terms of median copy numbers for selected cell types, we calculated 145 median protein copies for *E. coli*, 1888 median protein copies for *S. cerevisiae*, and 15,654 median protein copies for HeLa cells.

**Figure 7:**
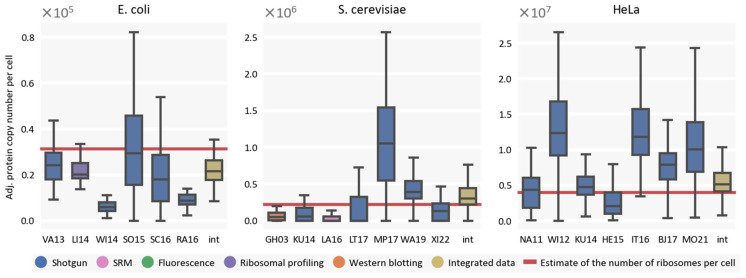
Distribution of adjusted copy numbers of ribosomal proteins in selected studies as well as in consensus proteomes. Adjusted protein copy number per cell denotes copy number of the ribosomal protein adjusted to the protein’s stoichiometry in the ribosome (see Section 4).

To provide an additional measure of the integrity of the resulting consensus proteomes, we explored how copy numbers of ribosomal proteins correlate with the published numbers of ribosome complexes per cell. Ribosomal proteins are produced in equimolar concentrations in the cell and their numbers are tightly regulated [45]. For *E. coli*, we assume an approximate number of 31,739 ribosomes per cell in selected conditions (see Section 4). For *S. cerevisiae*, a value of 220,000 ribosomes per cell has been reported [46]. Finally, for HeLa cells, differing values have been reported: 3.3 million [47], 4 million [37], and 9.5 million [48]. We selected a median of these values. Generally, median copy numbers of ribosomal proteins (adjusted to the ribosomal composition, see Section 4) in the integrated proteomes are close to the selected numbers of ribosomal complexes except for *E. coli*, which is a bit lower (Figure 7). We take this as an indication that the scale of our transformation is in agreement with supplementary quantitative data for these cell types.

However, it must be noted that the results of experiments which utilize mass spectrometry display considerable differences between copy numbers of ribosomal proteins. Lower copy numbers than expected can be attributed to some loss of material during sample preparation. However, considering the aforementioned phenomenon of tight balancing of the levels of ribosomal proteins [45], it is surprising to see ribosomal proteins with significantly higher copy numbers than expected. Nevertheless, it must also be taken into consideration that estimates of the number of ribosomes can noticeably vary in the literature (as is the case with HeLa). We expect that these results might prove to be stimulating to re-examine the levels of ribosomes and ribosomal protein copies in future studies.

## 3. Discussion

The present study explores the discrepancy in total protein copy numbers reported in whole proteome quantification studies. This discrepancy has been noticed before [12,49]. However, since then, more datasets have been published for common model organisms, such as the ones selected in our study, i.e., *E. coli*, *S. cerevisiae*, and HeLa cells. Exploring the results of 21 proteomic studies for these cell types, it seems likely that a variation in reported numbers is associated with subtle miscalculation performed at different steps of proteome quantification such as cell counting. First, selected datasets share proteins with the highest copy numbers per cell, which suggests that proteins not quantified in any study are present in low copies and would not have a large influence on the final result. Additionally, while we observe moderate levels of correlation for levels of individual proteins between the datasets, this factor alone cannot explain the observed difference in total protein copies per cell.

While there are more datasets than the ones used in this study, we prioritized datasets that performed original calculations of protein copy numbers. In fact, several published proteome quantification studies that have reported protein abundance in copies per cell used a presumed total number of protein copies for normalization [50,51]. While this is a reasonable approach, these studies used a lower total protein copy number than the one that can be calculated from the total protein mass per cell, as done in the present study. This is explained by the fact that previous publications that calculated the total number of protein copies per cell used different values of parameters, such as average protein mass [11], or performed a targeted quantification that did not include some of the highly expressed proteins in initial target selection [9]. Altogether, these observations highlight the importance of re-analysis and normalization of protein copy numbers. Our method of normalization of protein copies to a calculated total number of total protein copies per cell is based on total protein mass per cell and average protein mass. In the end, we obtained consensus proteome maps of *E. coli, S. cerevisiae*, and HeLa cells.

Additional attention should be paid to the HeLa proteome. Despite the fact that the HeLa proteome is supposed to be much more complex than proteomes of simpler organisms such as *E. coli* and *S. cerevisiae*, we observe similar, if not higher levels of correlation between individual datasets for HeLa. We believe that the reason for this is our data processing strategy that focused only on canonical proteins (master proteins [2]). Despite the fact that alternative splicing is known to diversify proteomes in higher eukaryotes, current mass spectrometry methods capture predominantly canonical proteins, which usually have the highest expression among all protein isoforms produced from one gene [52]. We reasoned that our canonical proteome map is a good enough consensus proteome that would be useful for most applications, but it is likely that, in the near future, isoform-resolved proteome maps of HeLa and other cells of higher eukaryotes will be published.

## 4. Materials and Methods

### 4.1. Data Processing

For all data processing steps, Python v.3.11 was used. Canonical proteome data for *E. coli*, *S. cerevisiae*, and HeLa were downloaded from UniProt (accessed on 17 August 2022). Data for absolute protein copy numbers per cell were extracted from a supplementary dataset for each of the selected whole proteome quantification studies. The exact supplementary datasets used are listed in Supplementary Table S1. For ID assignment, first, we considered whether a dataset contained UniProt IDs. If yes, we tested whether all UniProt IDs were present in downloaded proteomes from UniProt. In the case of protein groups, we contracted the protein group only to review canonical proteins. If the entry remained a protein group, it was excluded from further cross-study comparisons. Since there had been a change in nomenclature of several proteins since the publication of many of the selected studies, some UniProt IDs could not be assigned. To try and infer the UniProt ID, in these cases, we used B numbers for *E. coli* and ORF IDs for *S. cerevisiae*, if available, to infer UniProt IDs. If, in this case, the ID could not be assigned, the entry was excluded from cross-study comparisons and data integration. The results of ID assignment in terms of the proportion of correctly assigned IDs for each study are found in Supplementary Table S1.

### 4.2. Core Protein Assignment and Calculation of Pairwise Correlations

Proteins which were quantified in all of the selected datasets for a particular model organism were defined as the core proteins. Core 1 proteins were defined as proteins which were quantified in at least n − 1 dataset for a particular model organism. To perform calculations of the core, as well as pairwise Pearson’s correlation, only entries with successfully assigned UniProt IDs were considered. Pairwise Pearson’s correlations were calculated using a pearsonr function from the scipy.stats module. Heatmaps for correlations were created using a heatmap function from the seaborn module.

### 4.3. Normalization of Protein Copy Numbers in Individual Datasets

To calculate the total number of protein copies per cell from total protein mass per cell, we divided the literature-derived estimate of total protein mass per cell for each of the selected model organisms by the median average protein mass in the selected whole proteome quantification studies. Estimates of total protein mass per cell used were 280 ng for *E. coli* [6], 5.65 pg for *S. cerevisiae* [36], and 250 pg for HeLa cells, obtained as the average of values from several sources [18,37]. 

Next, the following formula was used for the calculation of the normalization factor for individual datasets:

f = (T × A × I × C)/S,

where T is the total number of protein copies per cell type as estimated from total protein mass per cell, A is the proportion of proteins in the dataset with successfully assigned UniProt IDs, I is the number of core proteins present in the dataset divided by total number of calculated core proteins for cell type, C is the minimal proportion of contribution of core proteins to total protein copy number across all studies which contributed to calculation of core for cell type, S is the sum of copies of proteins which belong to core in the dataset.

Normalized protein copy numbers in datasets were obtained by multiplication of original copy numbers by the dataset-specific normalization factor f.

### 4.4. Estimation of the Number of Ribosomes in E. coli in Selected Conditions

To estimate the number of ribosomes in cells of *E. coli* for the selected conditions, we used the results of Bakshi et al., who counted 27,000 ribosomes per µm^3^ on average in *E. coli* K-12 cells growing in rich medium (growth rate, µ = 1.11) [53]. For *E. coli*, it has been estimated that the mass fraction of ribosomal proteins increased almost linearly with growth rates of µ > 0.3 [54]. Accordingly, we calculated the number of ribosomes per µm^3^ for *E. coli* growing in minimal glucose medium (as is in most of the selected studies) by multiplying 27,000 by the relation of growth rates between our datasets and the Bakshi et al. study. For *E. coli* strain MG1655 cells growing in glucose minimal medium, a growth rate of 0.6 has been reported [41], therefore, we obtained a value of 14,595 ribosomes per µm^3^. Considering that *E. coli* strain MG1655 has been reported to have a volume of around 2.15 µm^3^ in corresponding conditions [17], we arrived at a final value of 31,739 ribosomes per cell.

### 4.5. Calculating Adjusted Copy Numbers of Ribosomal Proteins

To provide estimates of the number of ribosomes based on the data for copy numbers of individual proteins, it was required to account for organism-specific differences in the organization of ribosomes. First, in *E. coli* ribosomes, 50S ribosomal protein L7/L12 (UniProt ID P0A7K2) is present in 4 copies per ribosome [55]. Accordingly, we divided the copy numbers of this protein by 4 to calculate the distribution of ribosomal proteins for *E. coli* as a proxy for the number of ribosomes.

In *S. cerevisiae*, multiple ribosomal proteins are encoded by pairs of genes and differ slightly in the sequences of the resulting isoforms [56]. Accordingly, to provide estimates for the number of ribosomes based on the data for copy numbers of individual proteins, it was required to summarize copy numbers of such pairs of proteins to arrive at copy numbers of “ribosomal parts” rather than individual proteins. We performed summation according to our custom data based on the information from the UniProt database (Table S2).

In the case of HeLa cells, similar summations were performed only for RPS4X and RPS4Y proteins, which are the only alternative ribosomal proteins in human cytoplasmic ribosomes [57].

## 5. Conclusions

In conclusion, our study integrates multiple published protein abundance data for *E. coli*, *S. cerevisiae*, and HeLa cells. Despite significant differences in reported total protein copy numbers, we conclude that these datasets quantify dominant parts of the proteome, and thus, can be normalized to total protein mass per cell. Our calculations indicate that a typical *E. coli* cell contains around 6 million protein molecules, a *S. cerevisiae* cell contains approximately 80 million proteins, and a HeLa cell contains around 3.4 billion protein copies. In terms of protein copy density, it decreases from *E. coli* (which contains 2.5 million protein molecules per µm^3^) to *S. cerevisiae* (which has 2 million protein molecules per µm^3^) to HeLa cells (which have 1.5 million protein molecules per µm^3^). Our results generally agreed with some of the previously published estimates and, in the case of *S. cerevisiae*, improved the estimate of the total number of proteins, which was twice as low in some of the previous work. In general, we believe our integrated proteome datasets will be a useful resource for the scientific community.

## Figures and Tables

**Table 1:** Overview of selected proteomic studies of *E. coli*, *S. cerevisiae*, and HeLa.

Cell Type	Study	Code	Method	Strategy for Calculation of Protein Copies	Proteins Quantified	Total Protein Copies per Cell	Ref.
*E. coli*	Taniguchi et al., 2010	TA10	Fluorescence	Single-molecule fluorescence calibration	1018	94,571	[8]
	Valgepea et al., 2013	VA13	Shotgun MS	iBAQ, calibration with standards (UPS2) * and total protein per cell	1179	4,293,284	[14]
	Li et al., 2014	LI14	Ribosomal profiling	Relative protein synthesis rates multiplied by total protein per cell	3883	5,627,623	[10]
	Wisniewski et al., 2014	WI14	Shotgun MS	TPA and total protein per cell	2261	1,321,542	[15]
	Soufi et al., 2015	SO15	Shotgun MS	iBAQ, calibration with standards (UPS2) and cell count	1913	11,214,979	[16]
	Schmidt et al., 2016	SC16	Shotgun MS	LFQ, calibration with standards and cell count	2355	5,070,410	[6]
	Radzikowski et al., 2016	RA16	Shotgun MS	LFQ and total protein per cell	1959	2,220,410	[17]
*S. cerevisiae*	Ghaemmaghami et al., 2003	GH03	Western blotting	Calibration with standards and cell count	3868	46,664,471	[9]
	Kulak et al., 2014	KU14	Shotgun MS	TPA and total protein per cell	4570	48,114,163	[18]
	Lawless et al., 2016	LA16	SRM	Standards (QconCAT) and cell count	1167	56,322,039	[13]
	Lahtvee et al., 2017	LT17	Shotgun MS	iBAQ, calibration with standards (UPS2) and cell count	1788	96,407,334	[19]
	Martin-Perez et al., 2017	MP17	Shotgun MS	“Proteomic ruler”	3318	250,751,159	[20]
	Wang et al., 2019	WA19	Shotgun MS	“Proteomic ruler”	2582	71,802,810	[21]
	Xia et al., 2022	XI22	Shotgun MS	iBAQ, calibration with standards (UPS2) and total protein per cell	2526	73,823,343	[22]
HeLa	Nagaraj et al., 2011	NA11	Shotgun MS	iBAQ and total protein per cell	8078	2,007,666,667	[23]
	Wisniewski et al., 2012	WI12	Shotgun MS	TPA and total protein per cell (estimate)	8094	8,236,921,797	[24]
	Kulak et al., 2014	KU14	Shotgun MS	TPA and total protein per cell (estimate)	9677	2,982,812,197	[18]
	Hein et al., 2015	HE15	Shotgun MS	LFQ and total protein per cell	8804	2,916,903,614	[25]
	Itzhak et al., 2016	IT16	Shotgun MS	“Proteomic ruler”	8710	7,837,554,944	[26]
	Bekker-Jensen et al., 2017	BJ17	Shotgun MS	iBAQ and calibration with standards	14178	4,077,816,932	[5]
	Morgenstern et al., 2021	MO21	Shotgun MS	TPA and total protein per cell	8436	4,883,462,397	[27]

* Data in Valgepea et al., 2013 are based on rescaling of data from Arike et al., 2012 [28], who performed absolute quantification of *E. coli* cells using the iBAQ approach. Abbreviations: iBAQ—intensity-based absolute quantification; UPS2—universal protein standard 2; LFQ—label-free quantification; TPA—total protein approach; SRM—single reaction monitoring; QconCAT—quantification conCATamer

## Data Availability

The data presented in this study are available as Supplementary Materials.

## Supplementary Materials

The following supporting information can be downloaded at: https://www.mdpi.com/article/10.3390/ijms24032081/s1.
